# Associations between spouses’ oxytocin receptor gene polymorphism, attachment security, and marital satisfaction

**DOI:** 10.1371/journal.pone.0213083

**Published:** 2019-02-28

**Authors:** Joan K. Monin, Selin O. Goktas, Trace Kershaw, Andrew DeWan

**Affiliations:** 1 Social and Behavioral Sciences, Yale School of Public Health, New Haven, CT, United States of America; 2 Psychiatry, Yale School of Medicine, New Haven, CT, United States of America; 3 Chronic Disease Epidemiology, Yale School of Public Health, New Haven, CT, United States of America; Technion Israel Institute of Technology, ISRAEL

## Abstract

OXTR rs53576, a polymorphism on the oxytocin receptor gene, has previously been linked to individual differences in social behaviors. That is, individuals with the GG genotype show greater empathy, sociability, and emotional stability. In the context of close relationships, such psychological resources are associated with better relationship outcomes. However, no studies to our knowledge have examined associations between spouses’ OXTR polymorphisms, attachment security, and marital satisfaction. In the current study, 178 married couples (N = 356; ages 37–90) completed self-report measures of attachment security and marital satisfaction and provided saliva samples for genotyping. Results from Actor Partner Interdependence Models showed that individuals who had the GG genotype (actor effect) or had a spouse with the GG genotype (partner effect) reported greater marital satisfaction than individuals with AA or AG genotypes. Furthermore, greater attachment security mediated associations between GG genotype and marital satisfaction.

## Introduction

Attachment security, the feeling of emotional safety that comes from a history of close others being responsive to one’s needs, is associated with numerous benefits for married couples [1]. Attachment security plays an essential role in effective communication, problem solving, and social support [2,3] and it is a strong predictor of marital satisfaction over time [2]. While attachment security is thought to be influenced mainly by social experiences throughout the life course, a few studies have shown associations with genetic markers [4]. One such genetic marker is the oxytocin receptor (OXTR rs53576) polymorphism which has been linked with psychological resources more broadly, such as social bonding, empathy, sociability, and emotional stability [5]. Although past research has examined associations between genetic markers and attachment security [5,6], no study to our knowledge has examined whether the OXTR rs53576 genotype is associated with relationship outcomes and whether attachment security plays a role in this process. Furthermore, no study has examined these associations using dyadic data. The aim of this study was to examine among midlife and older adult married couples whether one’s own OXTR rs53576 genotype and one’s spouse’s OXTR rs53576 genotype are associated with marital satisfaction and whether these associations are mediated by attachment security.

### Oxytocin receptor gene and social and emotional skills

Oxytocin, a hormone produced in hypothalamus within the limbic system, has a central role in the modulation of social and emotional behaviors [7]. High oxytocin plasma concentrations have been implicated in the formation of social relationships such as maternal bonding, attachment, trust, and social affiliation [8]. Recent research on oxytocin has further revealed individual differences in psychosocial tendencies on a genetic level. The mechanism driving these differences is a single nucleotide genetic polymorphism in the oxytocin receptor gene named OXTR rs53576. Carrying a certain type of allele encoded as G and A has been shown to be the genetic marker of differences in social-emotional behavioral traits [9,10].

Being homozygous for the G allele is linked to having better social and emotional skills. For example, a meta-analysis examining the rs53576 polymorphism and sociality found that the G homozygotes responded to others around them more sociably as indicated by greater extraversion and empathy and lower social loneliness than people with AA or AG genotypes [11]. A recent study also showed that people with the GG genotype exhibit higher emotional stability which was associated with greater perceived social support and in turn general self-reported health (10). Notably, empathy and greater awareness of social cues have been consistently related to having a GG genotype [12–14].

Two of the core components of close relationships–trust and fairness have also been associated with OXTR. For example, one study showed that men with the GG genotype displayed more trusting behaviors than men with the A allele in an experiment in which two men participated in an investment game and lottery game measuring trust and risk behaviors, respectively [15]. Similarly, GG men were more trusting of others than AA men in a separate trust game experiment [16]. In another study, naïve observers were presented with silenced video clips of targets who were listening to their romantic partner disclosing a moment of suffering. The observers rated both male and female G homozygote targets as more prosocial in terms of compassion, trustworthiness, and kindness than AG or AA targets in the videos [17].

It has also been shown that individuals with at least one copy of the G (GG/AG) allele benefit more from receiving emotional support than those with the AA genotype. In an experiment in which participants were randomly assigned to receiving either no social support or to receiving social support before a stressful event, carriers of the G allele had lower cortisol levels and reported decreased anticipated stress compared to participants with the AA genotype [18].

Although there is now a substantial literature on OXTR rs53576 and its influence on how people respond in social situations, less is known about the association between OXTR rs53576 and close relationship outcomes. This is important because not only is one’s own genotype likely to influence one’s own thoughts, feelings, and behaviors, but close partners’ genotypes are likely to influence one’s thoughts, feelings, and behaviors. In the present study we focus on midlife and older adult spouses, as there tends to be high levels of interdependence in older adults in long term relationships [19].

### OXTR gene and romantic relationships

To our knowledge, the association between the OXTR rs53576 polymorphism and marital satisfaction is largely unknown. However, a small number of studies have examined the association between romantic relationship processes and OXTR. For example, one study investigated empathic communication in romantic couples combining five allelic variations of the OXTR single nucleotide polymorphisms (SNPs) into a single measure [20]. The study found that participants with a greater cumulative genetic risk on the five OXTR allelic variations displayed difficulties in empathic communication while interacting with their partner. In another study, twelve allelic variations of the OXTR SNPs were assayed to test whether they were associated with partner bonding behaviors among women. These self-reported partner bonding behaviors were assessed by the partner’s perception of closeness to their partner, their affectionate behaviors, marital stability, and reciprocity between two partners. Only one genotype rs7632287 was found to be associated with partner bonding [21]. Carriers of one or two copies of the A allele had lower levels of partner bonding behaviors and relationship quality as compared to carriers of the GG allele. In addition, the same women carrying the A allele were more likely to have had marital crisis within the past year. However, rs53576 in the same study did not influence partner bonding behaviors despite the evidence between rs53576 and social behavior.

### OXTR and attachment security

Adult attachment theory posits that the motivational system that helps explain how children bond with their parents early in life influences how people bond with their romantic partners as adults [22]. It is theorized that people develop different attachment styles, or cognitive representations of how responsive partners are, based on their interactions with their primary caregivers early in life and over time. There are two main attachment dimensions. Attachment anxiety reflects diminished self-worth, rejection sensitivity, and a tendency to engage in approval seeking behaviors. People high in the avoidance attachment dimension on the other hand avoid closeness with others in effort to maintain their independent self-worth [23]. People who are low in attachment anxiety and avoidance are considered high in attachment security. Attachment security is associated with greater marital satisfaction; whereas, attachment anxiety and avoidance are each associated with lower marital satisfaction [24–26].

Although attachment security is thought to be influenced by experiences with primary caregivers throughout the lifespan, it is also possible that genes lead to dispositions that make certain attachment styles more likely. A few studies have examined this possibility, but with mixed findings. The first of these studies explored the associations between genetic polymorphisms and attachment style as measured by the Experiences in Close Relationships Scale [23] in an undergraduate sample [6] and found no evidence that the rs53576 polymorphism on the OXTR gene was associated with attachment insecurity. However, in a study of mothers and toddlers, Bakermans-Kranenburg and colleagues (2008) found that OXTR rs53576 was related to maternal sensitivity, such that mothers with the AA/AG genotype were less sensitive than mothers with the GG genotype [27]. Similarly, Raby and colleagues (2013) found that the GG genotype of OXTR rs53576 was associated with continuity of attachment security from infancy to adulthood [28].

In addition to examining direct effects of the OXTR genotype, a number of studies have examined the gene by environment interaction model in which OXTR moderates the association between attachment style and behavioral outcomes. For instance, a sample of undergraduate couples in a sexual relationship were recruited for a study that examined the relationship between attachment security, predictors of disclosure, and genetic influences. Partners were each asked to answer questionnaires measuring their perceptions of the risks of disclosing to their partners, closeness, attachment security, in addition to providing saliva samples for genotyping. Partners who were insecurely attached and had the A allele were more likely to fear disclosing to their partners and perceived less emotional closeness to their partners than those with the GG genotype [29]. On the other hand, partners who were insecurely attached with the GG genotype feared disclosing to their partners less and perceived greater closeness than insecurely attached individuals who carried the A allele. Another investigation examined the associations between affective communication, attachment security, and the rs53576 polymorphism in a young adult sample who were involved in a sexual relationship. Findings indicated that even though participants high in attachment security displayed more affection towards their partners, the GG allele on rs53576 had a more pronounced effect for participants with low attachment security on affectionate communication [30].

Relatedly, OXTR rs53576 has also been shown to moderate the association between attachment security and psychopathology. People with low attachment security carrying the A allele tend to be more socially anxious [9]. Similarly, the risk of developing PTSD has been shown to be higher among U.S. Veterans with insecure attachment style and who are carriers of the A allele [9]. Researchers have suggested that the presence of the GG genotype buffers individuals from adverse life events.

### The present study

Aims of the present study were to examine associations between the OXTR rs53576 genotype, attachment security, and marital satisfaction among midlife and older adult married couples. We used secondary survey data from two existing experimental studies with different samples to examine the present aims. One experiment was designed to examine the effect of providing emotional support to an older or midlife adult spouse with chronic pain on the support-provider’s cardiovascular and self-reported emotional reactivity to watching the spouse do a household task. The other experiment was designed to examine whether support provision (no support, one-sided support, or mutual support) for older and midlife spouses’ health concerns affected each spouse’s cardiovascular and self-reported emotional reactivity. Survey instruments used for the present study were completed at a separate time (with filler activities in between or a separate visit) from the experimental tasks, minimizing the potential impact of carryover effects or priming related to providing or receiving support. We used the Actor Partner Interdependence Model to examine ‘actor effects’, the association between one’s own OXTR genotype and one’s own relationship outcomes, and ‘partner effects’, the association between one’s partner’s OXTR genotype and one’s own relationship outcomes [31]. As previous studies have shown significant associations between the OXTR GG genotype and positive relationship outcomes [20,21] and OXTR GG genotype and attachment security [29,30] among primarily younger adult individuals, we examined the following hypotheses with midlife and older adult married couples:

Hypothesis 1 (actor effect): One’s own GG genotype (compared to AA and AG) will be associated with one’s own greater marital satisfaction.
Hypothesis 2 (partner effect): One spouse’s GG genotype (compared to AA and AG) will be associated with the other spouse’s greater marital satisfaction.

Hypothesis 3a and 3b (actor effects): One’s own GG genotype (compared to AA and AG) will be associated with one’s own lower attachment anxiety and attachment avoidance.
Hypothesis 4a and 4b (partner effects): One spouse’s GG genotype (compared to AA and AG) will be associated with the other spouse’s lower attachment anxiety and attachment avoidance.
Hypothesis 5 (mediation effects; for both actor and partner effects): Lower attachment anxiety and avoidance will mediate the associations between GG genotypes and greater marital satisfaction.

Alternative Gene X Environment Hypothesis: Attachment anxiety and avoidance will moderate the associations between GG genotypes and marital satisfaction.

## Materials and methods

### Ethics statement

The present study was approved by Yale University’s Institutional Review Board (HIC # 1210011003 and 1103008149). Both spouses in each couple had to sign a consent form to participate in the study.

### Participants

Two study samples were combined to generate the combined sample for the present study. Study 1 consisted of 77 couples where both partners were at least 50 years of age and in which one partner had chronic pain. Study 2 consisted of 101 couples in which both partners were at least 50 years of age, with no eligibility restrictions regarding health status or chronic conditions. Thus, our total sample for the present study consisted of 178 heterosexual married couples (N = 356) living in the United States. See Table 1 for participant characteristics. Although Study 1 consisted of couples coping with chronic pain and Study 2 did not, we did not hypothesize that the genetic and attachment factors would be influenced by the presence of one spouse’s chronic pain. However, we tested for differences in all dependent and independent variables between the two samples to identify whether dummy study variables (categorical: Sample 1 = 1 or 0; Sample 2 = 1 or 0) should be included as covariates in the main models.

**Table 1:** Participant demographics.

Sample Characteristic	Wife *(N = 178)* *n %*		Husband *(N = 178)* *n %*	
Education^a^				
Did not complete high school	3	1.7	7	4.0
Completed high school	36	20.6	33	18.9
Postsecondary education/training	136	77.7	135	77.1
Employment^b^				
Employed	63	36.2	67	38.3
Homemaker	13	7.5		
Unemployed/retired	98	56.3	108	61.7
Income				
Less than $50,000	–	–	45	32.4
$50,000-$99,999	–	–	59	42.4
$100,000 or more	–	–	35	25.2
OXTR^c^				
AA	17	9.6	16	9.0
AG	62	47.8	71	39.9
GG	93	52.2	81	45.5
Hardy-Weinberg-Equilibrium^d^	X2 = 1.87, p = 0.171		X2 = 0.01, p = 0.938	
Race^e^				
White	169	96.6	164	93.7
Black	1	.6	2	1.1
Other	5	2.9	9	5.1
Children^f^				
Yes	132	77.2	136	78.6
No	39	22.8	37	21.4

^a^6 spouses did not report information on education.

^b^7 spouses did not report on employment status.

^c^6 wives and 10 husbands did not have genotyping results

^d^ Combined Hardy-Weinberg- Equilibrium (X2 = 1.03, p = 0.309)

^e^6 spouses did not report race.

^f^12 spouses did not report on children

### Procedure

#### Sample 1

Seventy-seven of the couples took part in a study about experiences with a spouse’s chronic pain condition. Individuals with musculoskeletal conditions (osteoarthritis, lower back pain) and their spouses were recruited from newspaper advertisements and community bulletins. To be eligible to participate, 1) the individual with pain had to be over 50 years old; 2) the couple had to be married or in a marriage-like relationship and have lived together for at least 6 months; 3) the spouse could not have a musculoskeletal condition that caused pain; and 4) if the spouse had another chronic condition that caused pain, the spouse had to have less pain on average than the individual with the condition. In addition, the spouse could not be taking a beta blocker, as one of the parent study outcomes was heart rate. The procedures consisted of a background questionnaire, saliva collection for genetic analysis, and a brief experiment in which the spouse watched the person with the condition perform a pain eliciting household task (i.e. carrying heavy groceries) in the lab while the spouse’s blood pressure and heart rate were monitored. For a detailed description of the procedure, see [32]. The background questionnaire was administered at the end of the laboratory session separately and privately after a 10-minute rest period. Each participant was asked to provide a mouth swab at the end of the visit.

#### Sample 2

The other 101 couples took part in a study about how spouses support each other with their health concerns. Participants were recruited from newspaper advertisements and community bulletins. To be eligible to participate, couples had to be married or in a marriage-like relationship, living together for at least 6 months, and both partners had to be at least 50 years old. Both spouses were excluded if at least one partner took beta-blockers as an outcome of the parent study was heart rate. The procedures of the study consisted of a background questionnaire that was completed at home. Participants were asked to complete the questionnaires separately and privately without sharing their answers. Participants also came in for a lab session in which they engaged in a set of discussions. This included a discussion of how they first met, a discussion about one of the wife’s health concerns, and a discussion about one of the husband’s health concerns. During the health concern discussions, couples were assigned randomly to (a) both support each other, (b) only the wife gave support, (c) only the husband gave support, or (d) neither gave support. At the end of the session, each participant was asked to provide a mouth swab.

### Measures

#### Marital satisfaction

Participants self-reported marital satisfaction using the 16-item Locke and Wallace Marital Adjustment Test (MAT) [33]. Several aspects of their relationship quality were measured: 1) their general level of marital happiness, using a scale from 1 (*very unhappy*) to 7 (*perfectly happy*) with one item; 2) agreement on 7 items capturing different relationship aspects (i.e., handling family finances, matters of recreation, friends, sex relations, etc.), using a scale from 1 (*always disagree*) to 6 (*always agree*); and 3) 7 questions on whether or not they give in when disagreements arise, their engagement in outside interests, and their views to current partner. The total scores are the sum of items, with the first 9 items unweighted and the last 7 items weighted as specified in a recent psychometric article using MAT [34]. The Cronbach alphas for the 7 items capturing agreement on relationship aspects was 0.93 for wives and 0.94 for husbands. Scores range from 28 to 116 among women, and from 26 to 119 among men. Wives’ marital satisfaction mean scores and standard deviations were M = 93.98 (SD = 17.09), respectively. Husbands’ marital satisfaction mean scores and standard deviations were M = 95.50 (SD = 16.30). The higher the MAT score, the higher level of marital satisfaction.

#### Attachment security

Participants rated their feelings about their relationship with their current romantic partner, using a modified 26-item version of the Experiences in Close Relationships Scale [23]. This measure assesses two dimensions of adult attachment: attachment anxiety (i.e., “I worry a lot about my relationship with my partner”) and attachment avoidance (i.e., “I am nervous when my partner gets too close to me”). Participants responded using a 7-point scale for each item (1 = disagree strongly; 7 = agree strongly). The anxiety score is calculated by taking the mean of items 2, 4, 6, 8, 10, 12, 14, 16, 18, 20, 22, 23, and 25. The avoidance score is the mean of the remaining items with 15, 19, and 24 reverse scored. Relationship-specific attachment was measured, which has been reported to be more accessible than trait attachment during interactions with romantic relationship partners [35]. The Cronbach’s alphas for attachment anxiety and attachment avoidance for men and women in each study ranged from 0.80 to 0.83. Wives’ attachment avoidance mean scores and standard deviations were M = 2.25 (SD = 1.03); for attachment anxiety they were M = 2.30 (SD = .89). Husbands’ attachment avoidance mean scores and standard deviations were M = 2.21, (SD = .99); for attachment anxiety they were M = 2.41 (SD = .99).

#### OXTR genotyping

Buccal swabs were collected using the Gentra Puregene Buccal Cell Kit (QIAGEN; Germantown, MD). Samples were stored at -20° C until used for DNA purification and genotyping. Purification of genomic DNA from the swab brushes was performed according to the manufacturer’s protocol. Purified DNA was then quantified using NanoDrop (Thermo Fisher Scientific; Waltham, MA). For genotyping, 5ng of DNA was arrayed per sample onto a 384-well plate and dried overnight. Genotyping of the OXTR polymorphism, rs53576, was performed with TaqMan assay assay number C___3290335_20 from Applied Biosystems (Foster City, CA) using a 5μl reaction volume. Plates were read on the CFX384 Real-Time PCR Detection platform (Bio-Rad; Hercules, CA). Samples were clustered automatically using CFX Manager software (Bio-Rad); the cluster discrimination plot was visually inspected, and any sample that fell outside of the genotype clusters was manually assigned to be a “no call”.

#### Covariates

We measured the following potential confounders: age, gender, ethnicity, education, income, whether or not each participant had at least one child, marriage length, and employment status.

### Statistical analyses

First, we examined correlations among all study variables. Next, we examined whether there were differences in study variables between Study 1 and Study 2 samples with t-tests and chi-square analyses. Second, to test models that included both partners’ GG variables (GG = 1; AA and AG = 0) predicting both partners’ marital satisfaction (Hypothesis 1 and 2) and attachment security (Hypothesis 3 and 4) and whether attachment security mediated associations between GG variables and marital satisfaction, we used a dyadic data analytic technique.

We used the Actor-Partner Interdependence Model (APIM) with structural equation modeling in Mplus 7.3, which employs full information maximum likelihood (FIML) estimation to use all available data [31]. When running each model, we first examined whether husbands and wives had distinguishable versus indistinguishable data (meaning that data did or did not systematically differ depending on the person’s role as a wife or a husband) by conducting Chi square difference tests between models where husbands’ and wives’ like effects were constrained to be equal or set to be independent. Chi-square difference tests for all models were 0, indicating tests were non-significant and that all husband’s and wives’ data was indistinguishable. All like paths for husbands and wives were constrained to be equal in each model. R-square values were calculated to determine the amount of variance that the predictors accounted for in the outcomes. We report three model fit indices: the confirmatory fit index (CFI), the Tucker Lewis Index (TLI), and the root mean squared error of approximation (RMSEA) [36]. For the CFI values of >.95, for the TLI values of >.90 [37], and for the RMSEA values of < .08 [37] reflect good fit of a specified model to the data.

Finally, to examine moderating effects of OXTR on the association between attachment dimensions and marital satisfaction, we ran mixed models in SPSS using APIM. Interaction terms between each attachment dimension and each OXTR actor and partner effect were entered into separate models predicting marital satisfaction.

## Results

### Correlation and chi-square analyses and the Hardy-Weinberg-Equilibrium

Spearman correlations were performed, and wives who were carriers of the GG genotype were less likely to have kids than the AA/AG wives to have children (*r* = -.158, *p* = .043). Wives’ children status also negatively correlated with the husbands’ genotype (*r* = -0.176, *p* = .026). Husbands’ children status and their reported income were found to be negatively correlated (*r* = -0.194, *p* = .022). There were no other significant associations between participant characteristics, which we measured as potential cofounders, on the one hand and genotype, marital satisfaction, or attachment dimensions for husbands or wives (ps>0.10). There were no significant differences in genotype (wives: *X*^*2*^ (1, *N* = 172) = 2.00, *p =* 0.655; husbands: *X*^*2*^ (1, *N* = 168) = 0.050, *p =* 0.824) or attachment dimensions (wives anxious attachment: t = -0.26, p = 0.770; wives avoidant attachment: t = 0.96, p = 0.337; husbands anxious attachment: t = 0.41, p = 0.682; husbands avoidant attachment: t = 2.04, p = 0.069) between Study 1 and Study 2. Also, across studies, chi square analyses showed that spouses’ genotypes were not significantly associated (*X*^*2*^ (1, *N* = 165) = 2.296, *p =* 0.130). Including the child status variable in all models did not significantly change the findings. In addition, the rs53576 genotypes were in the Hardy-Weinberg-Equilibrium for men and women and in the complete sample (see Table 1).

### Hypothesis 1 and 2: Did both partners’ GG genotypes relate to both partners’ marital satisfaction?

**Fig 1:**
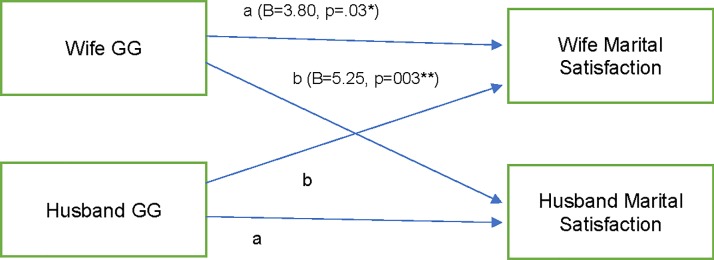
Structural equation model: Actor partner interdependence model with actor and partner gg genotypes predicting marital satisfaction. STD Standardized estimates. a = actor effect, b = partner effect. Letters indicate constrained paths. Also, in the model were associations between husbands’ and wives’ marital satisfaction (B = 104.75; p < .001) and husbands’ and wives’ GG genotype (B = 0.03, p = .13). Model accounts for 4% of the variance in husbands’ and wives’ marital satisfaction.

There was a significant actor effect such that when a person had the GG (and not AA or AG) genotype, their own marital satisfaction was greater (Fig 1). There was also a significant partner effect such that that when a person had the GG (not AA and AG) genotype, their spouse reported greater marital satisfaction. The model had good fit (RMSEA = 0.05; CFI = 0.98; TLI = 0.95; df = 2). The model accounted for 4% of the variance for husbands’ marital satisfaction and 4% for wives’ marital satisfaction.

### Hypothesis 3 and 4: Did both partners’ GG genotypes relate to both partners’ attachment security?

**Fig 2:**
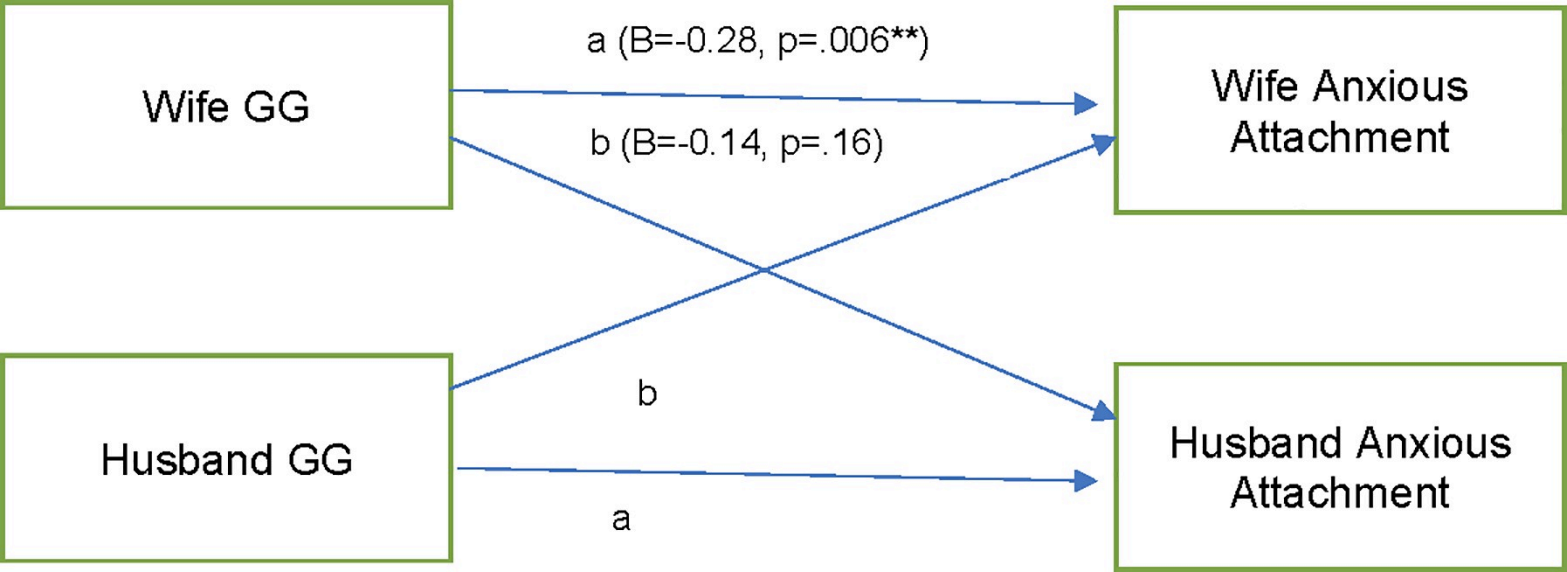
Structural equation model: Actor partner interdependence model with actor and partner GG genotypes predicting anxious attachment. STD Standardized estimates. a = actor effect, b = partner effect. Letters indicate constrained paths. Also, in the model were associations between husbands’ and wives’ anxious attachment (B = 0.04; p = .57) and husbands’ and wives’ GG genotype (B = 0.03, p = .13). Model accounts for 3% of the variance in husbands’ and wives’ anxious attachment.

**Fig 3:**
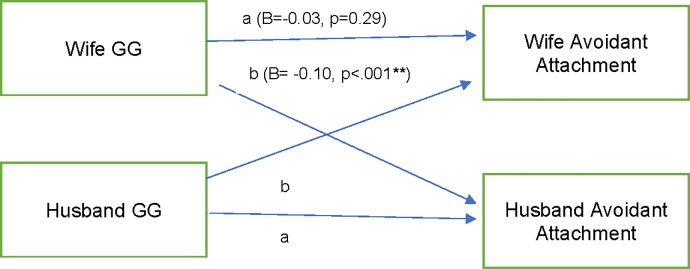
Structural equation model: Actor partner interdependence model with actor and partner GG genotypes predicting avoidant attachment. STD Standardized estimates. a = actor effect, b = partner effect. Letters indicate constrained paths. Also, in the model were associations between husbands’ and wives’ avoidant attachment (B = 0.31; p < .001) and husbands’ and wives’ GG genotype (B = 0.03, p = .13). Model accounts for 5% of the variance in husbands’ and wives’ anxious attachment.

There was a significant actor effect of GG on attachment anxiety, but the partner effect was not significant (model fit: RMSEA = 0.00; CFI = 1.00; TLI = 1.69; df = 2) (Fig 2). The model accounted for 3% of the variance for husbands’ anxious attachment and 3% for wives’ anxious attachment. There was a significant partner effect of GG on avoidant attachment; but the actor effect was not significant (model fit: RMSEA = 0.07, CFI = 0.90, TLI = 0.75; df = 2) (Fig 3). The model accounted for 5% of the variance for husbands’ avoidant attachment and 5% for wives’ avoidant attachment.

### Hypothesis 5: Did attachment security mediate associations between GG genotypes and marital satisfaction?

#### Anxious attachment

**Fig 4:**
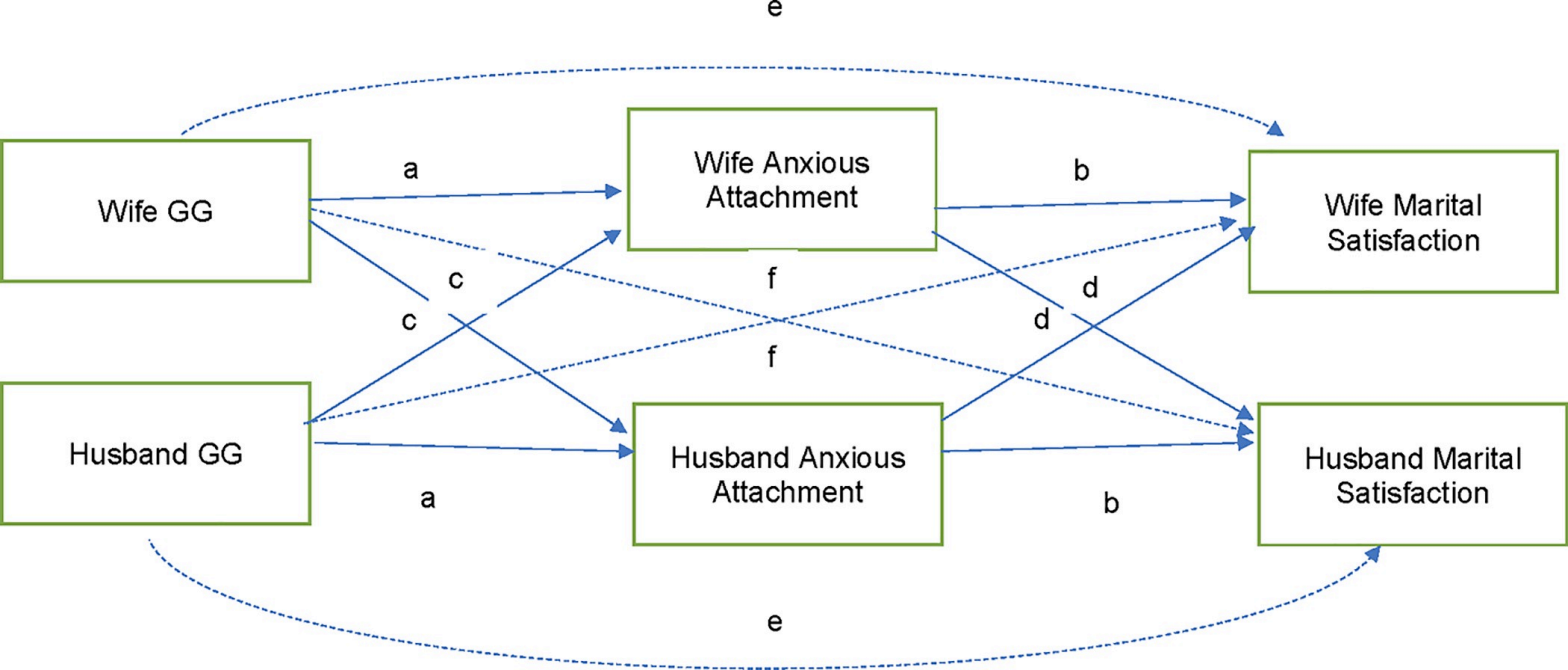
Structural equation model: APIM testing whether anxious attachment mediated association between GG and marital satisfaction. Letters indicate constrained paths tested in the model.

As shown in Table 2 and Fig 4, actor anxious attachment mediated the association between actor GG genotype and actor marital satisfaction (model fit: RMSEA = 0.00, CFI = 1.00, TLI = 1.04; df = 6). In other words, the wife’s lower anxious attachment mediated the association between the wife’s GG genotype and the wife’s greater marital satisfaction, and the same was true for husbands. The model accounted for 13% of the variance for wives’ marital satisfaction and 16% for husbands’ marital satisfaction.

**Table 2:** STD standardized structural equation model estimates: Actor partner interdependence model testing whether anxious attachment mediated association between GG and marital satisfaction.

	Estimate	SE	Est./SE	p
Path a: Actor GG → Actor Anxiety	-0.08	0.03	-2.78	0.005
Path b: Actor Anxiety → Actor Mar Sat	-4.77	0.89	-5.38	<0.001
Path c: Partner GG → Actor Anxiety	-0.05	0.03	-1.62	0.106
Path d: Partner Anxiety → Actor Mar Sat	-3.13	0.89	-3.53	<0.001
Path e: Actor GG → Actor Mar Sat	1.97	1.70	1.16	0.245
Path f: Partner GG → Actor Mar Sat	3.66	1.69	2.16	0.031
Other Associations Included				
Wife GG with Husband GG	0.02	0.02	1.20	0.23
Wife Anxiety with Husband Anxiety	0.07	0.07	0.99	0.32
Wife Mar Sat with Husband Mar Sat	78.22	18.85	4.15	<0.001

#### Avoidant attachment

**Fig 5:**
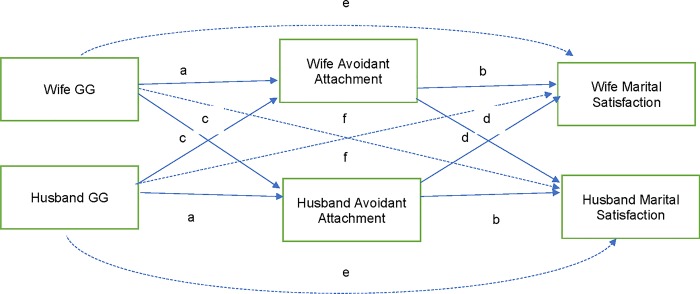
Structural equation model: APIM testing whether avoidant attachment mediated association between GG and marital satisfaction. Note. Letters indicate constrained paths tested in the model.

As shown in Table 3 and Fig 5, actor avoidant attachment mediated the association between partner GG and actor marital satisfaction (model fit: RMSEA = 0.06, CFI = 0.98, TLI = 0.96; df = 6). In other words, the wife’s lower avoidant attachment mediated the association between the husband’s GG genotype and the wife’s greater marital satisfaction. Likewise, the husband’s lower avoidant attachment mediated the association between the wife’s GG genotype and the husband’s greater marital satisfaction. The model accounted for 35% of the variance for wives’ marital satisfaction and 38% for husbands’ marital satisfaction.

**Table 3:** STD Standardized structural equation model estimates: Actor Partner interdependence model testing whether avoidant attachment mediated association between GG and marital satisfaction.

	Estimate	SE	Est./SE	p
Path a: Actor GG → Actor Avoid	-0.18	0.11	-1.71	0.087
Path b: Actor Avoid → Actor Mar Sat	-8.65	0.73	-11.90	0.001
Path c: Partner GG → Actor Avoid	-0.43	0.11	-4.06	<0.001
Path d: Partner Avoid → Actor Mar Sat	-2.46	0.73	-3.36	0.001
Path e: Actor GG → Actor Mar Sat	1.06	1.50	0.71	0.478
Path f: Partner GG → Actor Mar Sat	1.11	1.48	0.75	0.453
Other Associations Included				
Wife GG with Husband GG	0.03	0.02	1.49	0.14
Wife Avoid with Husband Avoid	0.27	0.08	3.51	<0.001
Wife Mar Sat with Husband Mar Sat	44.60	13.81	3.23	0.001

### Exploratory Hypothesis: Did attachment security moderate the associations between GG genotypes and marital satisfaction?

Mixed models in SPSS with APIM were performed to examine the interactions between each attachment dimension and actor and partner GG genotype predicting marital satisfaction. There were no significant interactions (see Table 4).

**Table 4:** SPSS mixed models examining interactions between attachment dimensions and GG predicting marital satisfaction using APIM.

	beta	SE	df	t	p
**Model 1: Attachment anxiety**					
Intercept	100.85	10.91	166.67	9.2	.000
Role	1.37	1.43	156.01	.96	.338
Actor GG	4.82	7.21	271.39	.67	.504
Partner GG	9.77	7.21	270.83	1.3	.177
Actor Anx	-3.25	3.66	172.13	-.88	.376
Partner Anx	-0.96	3.66	171.75	-.78	.793
Actor GG* Partner GG	-0.53	4.13	153.99	-.13	.898
Actor Anx * Partner Anx	-0.31	1.36	154.03	-.22	.819
Actor Anx * Actor GG	-0.45	1.98	257.34	-.22	.822
Actor Anx * Partner GG	-0.59	1.96	260.21	-.30	.765
Partner Anx * Actor GG	-0.42	1.97	265.61	-.21	.832
Partner Anx * Partner GG	-1.85	1.99	262.84	-.93	.354
**Model 2: Attachment avoidance**					
Intercept	102.99	7.49	177.97	13.74	.000
Role	0.85	1.31	155.99	.644	.520
Actor GG	7.25	5.19	290.11	1.40	.164
Partner GG	2.32	5.18	286.49	.45	.653
Actor Avoid	-4.17	2.47	198.60	-1.69	.093
Partner Avoid	3.22	2.44	190.41	1.32	.189
Actor GG* Partner GG	-0.47	3.41	153.95	-.14	.892
Actor Avoid * Partner Avoid	-1.64	.750	153.98	-2.19	.030
Actor Avoid * Actor GG	-1.17	1.61	293.96	-.73	.466
Actor Avoid * Partner GG	1.27	1.59	290.57	.80	.427
Partner Avoid * Actor GG	-1.37	1.60	298.73	-.86	.392
Partner Avoid * Partner GG	-1.70	1.60	294.79	-1.05	.293

Anx = Attachment anxiety; Avoid = Attachment avoidance

## Discussion

The present study tested the overarching hypothesis that the OXTR genotype would have both actor and partner associations with self-reported marital satisfaction among midlife and older married couples and that attachment security would be a pathway. Supporting our hypothesis, we found a significant association between one’s own GG genotype and one’s own greater marital satisfaction. We also found a significant association between one spouse’s GG genotype and the other spouse’s greater marital satisfaction. In other words, having at least one spouse in a marriage who has the GG genotype is associated with greater marital satisfaction for each spouse.

Also, as hypothesized, we found that lower actor anxious attachment mediated the association between actor GG genotype and greater actor marital satisfaction. In other words, GG wives were less anxiously attached, and their lower anxious attachment was related to greater marital satisfaction. Likewise, GG husbands were less anxiously attached which benefited their marital satisfaction. This actor effect, not differentiated by gender, suggests that OXTR rs53576 marks a socially aware and responsive disposition that makes it more likely to form cognitive representations of others as being available and responsive. From there, being low in attachment insecurity, specifically anxious attachment, is associated with greater self-reported marital satisfaction. A large literature shows that being low in anxious attachment is associated with better relationship quality, probably because less anxiously attached individuals are less likely to be jealous and intrusive in their caregiving behaviors than more anxiously attached individuals [38].

We also found that lower actor avoidant attachment mediated the association between partner GG genotype and actor greater marital satisfaction. Thus, a wife’s GG genotype is related to lower avoidant attachment in the husband which leads to the husband’s greater marital satisfaction. This is also the case for a husband’s GG genotype relating to lower avoidant attachment in the wife which leads to the wife’s greater marital satisfaction. This is an intriguing finding in that one spouse’s socially adaptive disposition may be shaping the other spouse’s attachment security and in turn their marital satisfaction. As these are midlife and older married couples who on average have been together for decades, it is possible that the genes of one partner play out in relationship behaviors that slowly influence their partner’s experience in the relationship over time. However, because this was a cross-sectional study, we cannot be certain about causal influences of one partner’s genes on the other partner’s attachment security.

We did not find that attachment anxiety or avoidance moderated the associations between actor and partner GG genotypes and reports of marital satisfaction. Instead of providing evidence for a gene X environment model [39], we found that having the GG polymorphism of the OXTR rs53576 had a direct effect or served as a marker. To be exact, we found that one’s genetic polymorphism reflected a socially competent and empathetic disposition that is likely to lead to secure attachment which in turn leads to greater marital satisfaction.

We also did not find gender differences in this study, which is in line with the findings of most studies of OXTR rs53576 and psychological and relationship quality outcomes. Thus, it seems that regardless of one’s role as a husband or wife, there may be genetic influences that make it more or less likely for both partners to be satisfied. It also suggests that genetic markers may be more important in the context of close relationship functioning than gender roles learned from one’s environment over the life course.

Strengths of this study include the use of dyadic data and analysis, the midlife and older adult community samples, and the inclusion of an interpersonal process mediator. A limitation of this study is the lack of racial and socioeconomic diversity of the sample which affects generalizability. Generalizability of the findings may also be affected by the fact that data included one sample in which one member of the couple had chronic pain. In addition, both samples originated from data in which married spouses were instructed to focus on health concerns and either provide or receive emotional support from their partner. Although the survey instruments were completed at a separate time from the experimental support tasks, it is possible that this context may have affected participants’ attachment and marital satisfaction ratings.

In conclusion, this study is the first to our knowledge to examine associations between one’s own OXTR genotype and one’s spouse’s OXTR genotype and how both spouses’ genetic polymorphisms relate to the quality of the marriage. Results of this study suggest that having at least one spouse in a marriage with an OXTR GG genotype is associated with both partners feeling satisfied and this is because spouses feel more securely attached to one another.

## Supporting information

S1 Dataset (SAV)
